# Active-Site Models of *Streptococcus pyogenes* Cas9 in DNA Cleavage State

**DOI:** 10.3389/fmolb.2021.653262

**Published:** 2021-04-21

**Authors:** Honghai Tang, Hui Yuan, Wenhao Du, Gan Li, Dongmei Xue, Qiang Huang

**Affiliations:** ^1^State Key Laboratory of Genetic Engineering, Shanghai Engineering Research Center of Industrial Microorganisms, Ministry of Education Engineering Research Centre of Gene Technology, School of Life Sciences, Fudan University, Shanghai, China; ^2^Multiscale Research Institute of Complex Systems, Fudan University, Shanghai, China

**Keywords:** Gene editing, CRISPR-Cas9, DNA cleavage mechanism, Molecular dynamics, off-target effects

## Abstract

CRISPR-Cas9 is a powerful tool for target genome editing in living cells. Significant advances have been made to understand how this system cleaves target DNA. However, due to difficulty in determining active CRISPR-Cas9 structure in DNA cleavage state by X-ray and cryo-EM, it remains uncertain how the HNH and RuvC nuclease domains in CRISPR-Cas9 split the DNA phosphodiester bonds with metal ions and water molecules. Therefore, based on one-and two-metal-ion mechanisms, homology modeling and molecular dynamics simulation (MD) are suitable tools for building an atomic model of Cas9 in the DNA cleavage state. Here, by modeling and MD, we presented an atomic model of SpCas9-sgRNA-DNA complex with the cleavage state. This model shows that the HNH and RuvC conformations resemble their DNA cleavage state where the active-sites in the complex coordinate with DNA, Mg^2+^ ions and water. Among them, residues D10, E762, H983 and D986 locate at the first shell of the RuvC active-site and interact with the ions directly, residues H982 or/and H985 are general (Lewis) bases, and the coordinated water is located at the positions for nucleophilic attack of the scissile phosphate. Meanwhile, this catalytic model led us to engineer new SpCas9 variant (SpCas9-H982A + H983D) with reduced off-target effects. Thus, our study provides new mechanistic insights into the CRISPR-Cas9 system in the DNA cleavage state, and offers useful guidance for engineering new CRISPR-Cas9 editing systems with improved specificity.

## Introduction

The RNA-guided CRISPR-Cas9 nuclease from *Streptococcus pyogenes* (SpCas9) has been widely used as a powerful and versatile tool for genome engineering (Hsu et al., 2014; Wang et al., 2016; Chen and Doudna, 2017). Guided by a pre-designed sgRNA with a 20-base long sequence for targeting DNA, SpCas9 could cleave corresponding complementary sequences in the genome through RNA: DNA hybridization (Jinek et al., 2012; Cong et al., 2013). Many studies have demonstrated that this cleavage process includes: first, the Recognition lobe (REC) domains of SpCas9 interact with the common sgRNA scaffold to form an Ribonucleoprotein (RNP) complex for recognizing the N is any one of bases (Adenine, Thymine, Cytosine, or Guanine), G is Guanine (NGG) motif just downstream the 20-mer targeting sequence, and then mediates the formation of the RNA: DNA heteroduplex; next, the HNH and RuvC endonuclease domains catalyze the hydrolysis of two DNA phosphodiester bonds in the complementary and non-complementary strands, respectively (Anders et al., 2014; Jinek et al., 2014; Nishimasu et al., 2014; Jiang et al., 2015, 2016). Although significant advances have been made in the past years, a complete understanding of the DNA hydrolysis mechanisms of the HNH and RuvC active sites, especially those of the RuvC domain is still lacking.

Structural and biochemical studies have suggested that the catalytic residues in the HNH active sites are D839, H840, and N863, which may employ the one-metal-ion mechanism to hydrolyze the complementary strand of the target DNA (Jinek et al., 2012; Nishimasu et al., 2014), in agreement with recent research (Zhu et al., 2019). Also, some studies reported that D10, E762, H983, and D986 are the catalytic residues of the RuvC domain, and then suggested that they utilize the two-metal-ion hydrolysis mechanism to split the non-complementary (non-target) strand (De Vivo et al., 2008; Ho et al., 2010; Nishimasu et al., 2014; Palermo et al., 2015; Chen and Doudna, 2017). However, unlike those of the HNH residues, the precise roles of these RuvC residues in the DNA cleavage remain debated. For example, for the HNH domain, it has been firmly established that H840 acts as the general (Lewis) base to activate the water molecule for nucleophilic attack of the scissile phosphate (Nishimasu et al., 2014). For the RuvC domain, in contrast, several views considered that H983 is the general base for the DNA hydrolysis (Zuo and Liu, 2016; Chen and Doudna, 2017), and others proposed that this residue plays a role in coordinating the metal ion (Fernandes et al., 2016). To clarify such mechanistic problems, direct structural information about the wild-type HNH and RuvC active-sites in the DNA cleavage state is critical. So it becomes very important to obtain the cleavage-activating structure of SpCas9 in complex with sgRNA and the target DNA.

Unfortunately, similar to structural studies of many enzymes, it is currently difficult to use the wild-type, active SpCas9 protein to form a stable complex with a full-length target DNA; alternatively, activity-dead mutants were often used for the structural determination (Anders et al., 2014; Jinek et al., 2014; Nishimasu et al., 2014; Jiang et al., 2016; Huai et al., 2017). Probably due to such a technical limitation, available x-ray crystal structures of the mutants were often resolved in the cleavage-inactive states, in which the HNH active-site was found far from the scissile bond of the complementary strand (>13 Å) (Nishimasu et al., 2014; Jiang et al., 2016). Also, in most of the resolved structures, certain numbers of nucleotides from the 20-mer non-target sequence are missing (Anders et al., 2014; Jiang et al., 2016). These made it difficult to build atomic models in the DNA cleavage state. To address this problem, we previously used cryo-EM to capture the active conformations of SpCas9, and obtained a structure of the SpCas9–sgRNA–DNA ternary complex in which the HNH active-site is nearest to the split bond of the complementary strand among all the available structures (Huai et al., 2017). However, this structure did not enable us to accurately build atomic models for the wild-type active-sites with metal ions and water molecules. Even though the current studies have obtained two cryo-EM structures (6O0Y and 6VPC) around 3.3 Å, the atomic models of their active sites in the cleavage (catalytic) state cannot be completely and accurately built due to their HNH and RuvC domains are not in the cleavage (catalytic) state at the same time: among them, the conformation of the HNH active sites in 6O0Y resembles catalytic state (Zhu et al., 2019), whereas the conformation of the RuvC active sites in 6VPC is in the catalytic state (Zhu et al., 2019; Lapinaite et al., 2020). Thus, how the HNH and RuvC active sites in a sharing structure organize with Mg^2+^ ions and water molecules to hydrolyze the DNA phosphodiester bonds remains open.

Here, we further refined the cryo-EM structure of the SpCas9–sgRNA–DNA ternary complex to be in the DNA cleavage state, and thereby rebuild the atomic model for the active complex by molecular modeling according to one- and two-metal-ion mechanisms. For this, we will present the model of the wild-type HNH and RuvC active-sites in complex with DNA, Mg^2+^ ions, and water molecules, and obtain a refined cryo-EM structure of SpCas9 in DNA cleavage state. Meanwhile, our mechanistic models indicate that the residues D10, E762, H983, and D986 are in the first coordination shell of the RuvC active-site with the ions, suggesting that H983 coordinates with the Mg^2+^ ion, and that the general base for the RuvC catalysis is likely H982 or/and H985.

## Results and Discussion

### Atomic Models of Nuclease Active-Sites

SpCas9 can cleave DNA by RNA guiding and has developed a powerful genome-editing tool that is widely used in many fields (Hsu et al., 2014; Wang et al., 2016; Chen and Doudna, 2017). To understand the conformational changes of SpCas9 in the atomic level and its interaction with nucleic acids, researchers used x-ray or cryo-electron microscopy to resolve SpCas9 structures with various forms, including SpCas9 monomer (4CMP) (Jinek et al., 2014), SpCas9–sgRNA binary complex (4ZT0) (Jiang et al., 2015), and SpCas9–sgRNA–DNA ternary complexes (4OO8, 4UN3, 5F9R, 5Y36, 6O0Y, and 6VPC) (Anders et al., 2014; Nishimasu et al., 2014; Jiang et al., 2016; Huai et al., 2017; Zhu et al., 2019; Lapinaite et al., 2020; Supplementary Figure 1). However, the aforementioned atom models are not all-atom structures of SpCas9 in the DNA shearing-active state and are thereby limitations of our understanding of the precise roles of the critical sites in the SpCas9 catalytic centers, especially the key sites in the RuvC active center. To overcome the difficulties of resolving an active structure with a high resolution for elucidating the precise roles of the key sites in the RuvC catalytic center, based on the currently available structures of SpCas9, through homology modeling, and molecular dynamics simulation, we intended to rebuild a model of SpCas9–sgRNA–DNA ternary complex in the DNA cleavage state from theories.

**FIGURE 1:**
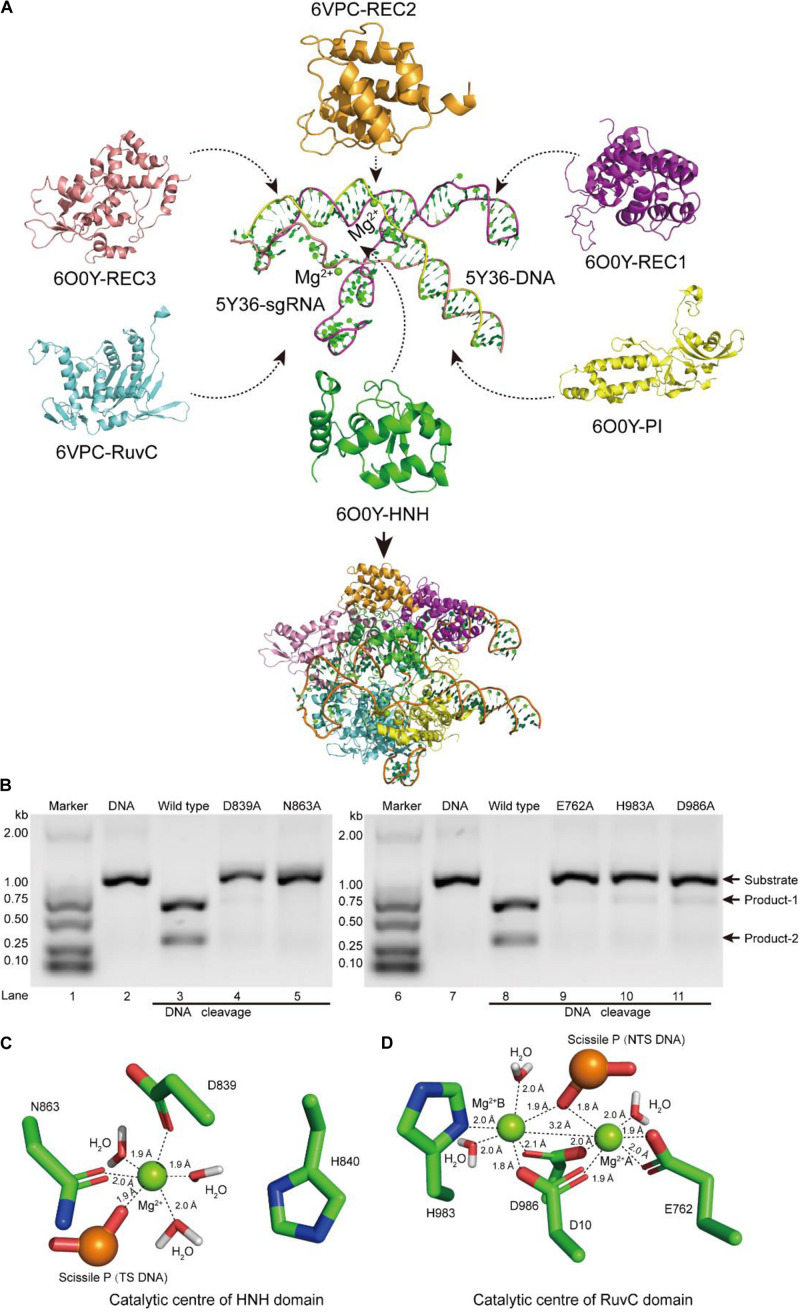
Atomic model of the ternary complex in the DNA cleavage state. **(A)** The structural assembly of SpCas9–sgRNA–DNA ternary complex. Domains HNH, REC1, REC3, and PI come from atomic model 6O0Y, and domains REC2 and RuvC derive from atomic model 6VPC. **(B)** *In vitro* cleavage activities of SpCas9 and its mutants. These mutation sites are located in the HNH and the RuvC domains, respectively. The target DNA molecules are cleaved into two products (product-1 and -2). **(C,D)** The Mg^2+^ ion coordination of the catalytic centers (HNH and RuvC, respectively). The distances between ligands and corresponding Mg^2+^ ions are about 2.0 Å.

To screen out suitable atom models as the structural templates for constructing SpCas9 in shearing active state, we aligned and analyzed the protein structures of the SpCas9–sgRNA–DNA ternary complexes (Anders et al., 2014; Nishimasu et al., 2014; Jiang et al., 2016; Huai et al., 2017; Zhu et al., 2019; Lapinaite et al., 2020), and observed that the structural orientations of SpCas9 were highly consistent below the oblique axis, while those above the oblique axis were slightly different (Supplementary Figure 2). This indicated that any single atomic model among SpCas9 structures can also be selected as a template to build the structure of REC1 and PI domains, but the construction of REC2, REC3, HNH, and RuvC domains needs to be analyzed and screened further. Considering the structural characteristics of these three atomic models (5Y36, 6O0Y, and 6VPC) (Huai et al., 2017; Zhu et al., 2019; Lapinaite et al., 2020) in the existing structures, they are the only active structures of SpCas9 so far. Among them, 5Y36 has magnesium ions in the catalytic centers and the atomic structure of the full-length DNA, while 6O0Y and 6VPC displayed the catalytic states of the HNH and RuvC domains in the DNA cleavage, respectively. Therefore, we presumed that SpCas9–sgRNA–DNA ternary complex in the DNA cleavage state might be rebuilt from theories, through selecting these three atomic models as references and using complementary strategies among their structures. First, we aligned these three structures and saved the coordinates of their relative positions; Next, we extracted atomic coordinates of nucleic chains and magnesium ions from 5Y36 (Huai et al., 2017); Subsequently, we transferred the atomic coordinates of REC2 and RuvC domains in 6VPC and the atomic coordinates of REC1, REC3, PI, and HNH domains in 6O0Y to the aforementioned nucleic chains (Zhu et al., 2019; Lapinaite et al., 2020). Finally, these structural fragments were assembled into a complete-initial structure of the SpCas9–sgRNA–DNA ternary complex (Figure 1A). To obtain the structure of ternary complex in the active state, the aforementioned ternary complex was accommodated into two cryo-EM densities maps (end-21308.map and end-0584.map, respectively) (Zhu et al., 2019; Lapinaite et al., 2020), which was processed by Situs-3.1 and Rosetta programs (Ashworth and Baker, 2009; Wriggers, 2010). This result showed that secondary structural elements in the ternary complex matched well into the EM density maps (Supplementary Figure 3). And in this active structure of the ternary complex, we observed that the locations of the key sites in the HNH and RuvC catalytic centers were consistent with those of 6O0Y and 6VPC (Supplementary Figure 4; Zhu et al., 2019; Lapinaite et al., 2020), respectively. In addition, in the two-metal center, the Nδ atom of H983 points toward the Mg^2+^ ion A, thus H983 may have a potential ability to stabilize the Mg^2+^ ion A (Supplementary Figure 5), rather than a general. Thus these data suggested that we successfully refined the structure of SpCas9 in the active state by the structural-complementary strategy.

In our atom model, we observed that the key sites of HNH and RuvC active centers, including residues D839, N863, E762, H983, and D986, embraced Mg^2+^ ions, consistent with the previous report (Nishimasu et al., 2014). Therefore, the result suggested that these residues are critical sites of the active centers in SpCas9, which is supported by an alanine scan. As shown in Figure 1B, alanine (Ala) substitution of D839, N863, E762, H983, or D986 may convert the wild-type SpCas9 into a nickase. Compared with the wild-type (lanes 3 and 8 in Figure 1B), the cleavage activities of the mutants D839A and N863A are sharply reduced and disappeared, respectively (lanes 4 and 5 in Figure 1B). Likewise, the cleavage activities of the mutants E762A, H983A, and D986A are significantly weakened (lanes 9, 10, and 11 in Figure 1B). Thus, these residues are essential for binding the metal ion and catalyzing the target DNA. However, we did not know whether this model is a catalytic state in DNA cleavage. To obtain the atomic model of SpCas9 in the DNA catalytic cleavage state, based on the one- and two-metal principles (Steitz and Steitz, 1993; Yang, 2008), we fixed magnesium ions and the coordination residues around them to resemble an octahedral symmetry, and performed molecular dynamics (MD) simulation for this. With coordination fixation, we set up coordination distances (near 2.0 Å) between magnesium ions and the corresponding coordination residues in the catalytic centers. After MD, in the HNH catalytic center, we observed that an Mg^2+^ ion is surrounded by D839 and N863, water molecules, and the phosphate group (Figure 1C). The Mg^2+^ ion coordinates with the Oδ atoms of D839 and N863, the O atoms of water molecules and the phosphate group; and the coordination geometry between the Mg^2+^ ion and the oxygen atoms is a near octahedral symmetry with the ligand-to-mental ion distance between 1.8 and 2.0 Å (Figure 1C); these features are almost consistent with those of one-metal-ion model (Yang, 2008). Meanwhile, the Nδ atom of H840 in the second structural shell points toward the corresponding phosphate group (Figure 1C), and could act as the general base to activate the water molecule for the nucleophilic attack on the phosphodiester bond. Similarly, in the RuvC catalytic center, two Mg^2+^ ions are enclosed by D10, E762, D986, and H983, water molecules, and the phosphate group (Figure 1D). In line with the two-metal-ion active-site model (Steitz and Steitz, 1993; Nowotny and Yang, 2006; Yang, 2008), these two Mg^2+^ ions are about 3.2 Å apart, and jointly coordinate with the Oδ atoms of D10, E762, and D986, the Nδ atom of H983, and the O atoms of water molecules and the phosphate group (Figure 1D). The distance between the ligands and corresponding Mg^2+^ ion is about 2.0 Å, which forms two near octahedrons (Figure 1D). At the same time, in this active state, the Root mean square deviation (RMSD) of the ternary complex was minor and stable, approximately 3 Å (Supplementary Figure 6). Thus, we succeed in refining the atomic model of the SpCas9 in cleavage (catalytic) state and revealing the potential catalysis roles for the key sites of the HNH and RuvC catalytic centers in hydrolyzing DNA.

### Catalytic Role of RuvC Residue H983

Previous studies have shown that the active sites of the RNase H [Protein data bank (PDB) ID: 1ZBL] consist of four conserved residues (D71, E109, D132, D192), and coordinate with two metal ions (Nowotny and Yang, 2006). Meanwhile, earlier studies also revealed that the function of the RNase H is similar to those of the RuvCs (PDB IDs: 4LD0, 1HJR, and 4UN3), and mainly cleaves nucleic acid chains by the two-metal-ion mechanism (Ariyoshi et al., 1994; Nowotny et al., 2005, 2007; Chen et al., 2013; Gorecka et al., 2013; Nishimasu et al., 2014). To investigate the catalytic role of H983 in the RuvC active center, we performed structure superpositions on the above four PDBs. In their catalytic domains, aside from the secondary structures (αββ), the orientations of these key catalytic residues are semblable, for example, site-1, site-2, site-3, and site-4 (D192, D138, H143, and H983) (Supplementary Figure 7). Therefore, the function of the residue H983 in the SpCas9 may be to coordinate with the Mg^2+^ ion A.

**FIGURE 2:**
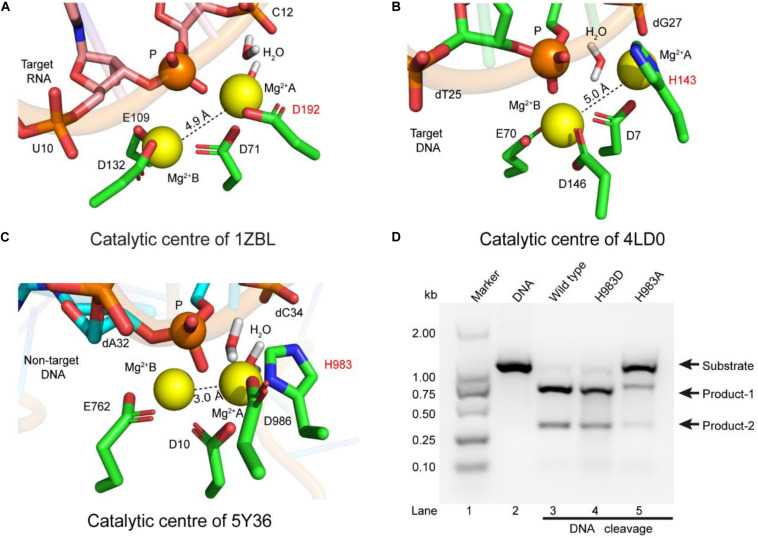
The coordination configurations of the catalytic centers. **(A–C)** The stereoviews of the two-metal catalytic centers in the atomic models (PDB IDs: 1ZBL, 4LD0, and 5Y36, respectively). The Mg^2+^ ions are coordinated by the ligands (amino acids, phosphate groups, and water molecules). **(D)** *In vitro* cleavage activities of SpCas9 and its mutants. The target DNA molecules are cleaved into two products (product-1 and -2).

To identify the catalytic role of H983 in the RuvC active site, we analyzed whether the conformations of residues H983 (5Y36), H143 (4LD0), and D192 (1ZBL) are consistent in the Mg^2+^ coordination by MD simulation after inserting Mg^2+^ into active centers (Figure 2). In the 1ZBL or the 4LD0, the distance between these two Mg^2+^ ions is about 5.0 Å; the scissile phosphorus is nearly located between the Mg^2+^ ions A and B; the D192 or H143 and at least one water molecule coordinate with the Mg^2+^ ion A (Figures 2A,B). Similarly, in the 5Y36, these two Mg^2+^ ions are spaced 3.0 Å apart; the Mg^2+^ ions A and B are bisected by the scissile phosphorus; the H983 and two water molecules coordinate with the Mg^2+^ ion A (Figure 2C). Thus, these results suggest that H983 of SpCas9 may be involved in immobilizing (anchoring) the Mg^2+^ ion A to maintain the reaction conformation of the catalytic center, consistent with the previous suggestion (Fernandes et al., 2016).

Since structure studies have shown that the action of the H983 in the SpCas9 is equivalent to that of the D192 in the RNase H, we presumed that the His mutation into Asp (H983D) at site 983 may retain the native cleavage activity; in contrast, the cleavage activity with the His mutation into Ala (H983A) will be weakened or may disappear. As expected, the cleavage activity of the mutant H983D is similar to that of the wild-type SpCas9 (lanes 3 and 4 in Figure 2D), in agreement with the cleavage activity of the mutant H983N (data not shown); and that of the mutant H983A was sharply reduced (lane 5 in Figure 2D). Thus, these results demonstrate that H983 can stabilize the Mg^2+^ ion A during SpCas9 cleaving the DNA, rather than the general base as the previous deductions (Chen and Doudna, 2017; Palermo, 2019). Moreover, the distance analysis between the Mg^2+^ ions A and B in the SpCas9 and its mutants also supports this view (Supplementary Figure 8), because the substitution of H983 with Ala (A) or Asp (D) affects the stability of the Mg^2+^ ion A.

### Lewis (General) Bases of RuvC Active-Site

**FIGURE 3:**
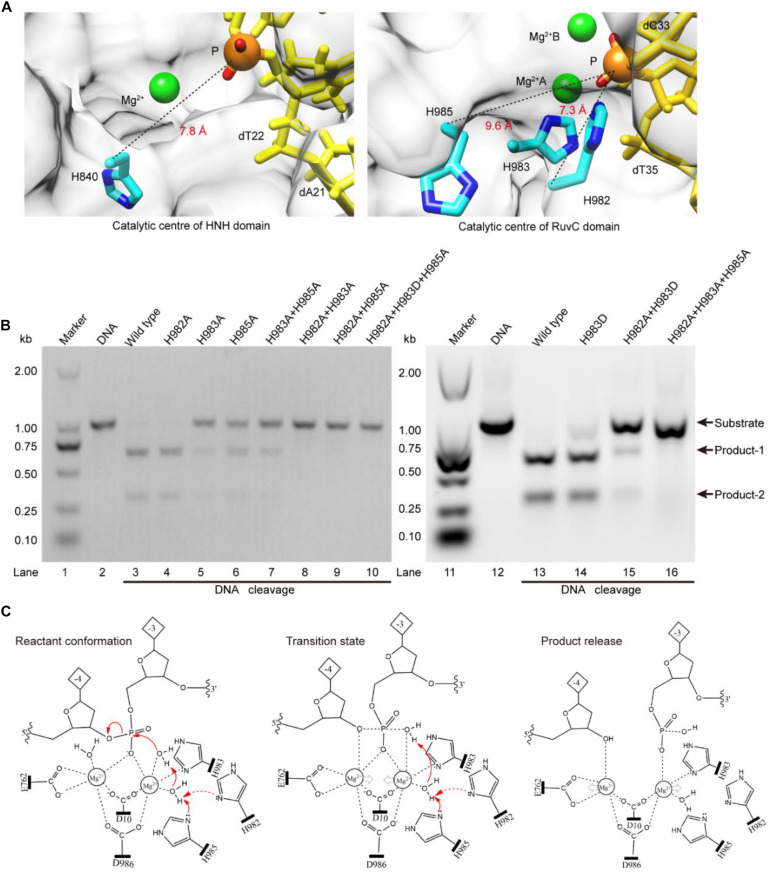
The Lewis bases in the SpCas9 catalytic centers. **(A)** The Lewis bases in the HNH and the RuvC. H840 is the Lewis base in the one-metal domain; H982 and H985 are the potential Lewis bases in the two-metal domain. **(B)** *In vitro* cleavage activities of the SpCas9 mutants. The target DNA molecules are cleaved into two products (product-1 and -2). **(C)** The two-metal-ion model of the RuvC domain. Reactant conformation: the cooperative motion of two metal ions. Transition state: the high-energy intermediate. Product release: the cleavage and breakage of the target DNA strands.

The above results indicate that the RuvC residue H983 coordinates with the Mg^2+^ ion, and is not a general base (Chen and Doudna, 2017; Palermo, 2019). Therefore, it might be other residues to serve as general bases during the RuvC domain hydrolyzing the No-target strand (NTS). To confirm this, referring to the distance (7.8 Å) between the C_α_ atom of the general base H840 and the corresponding scissile phosphorus, we analyzed residues around the scissile phosphorus in the RuvC catalytic center. As shown in Figure 3A, we observed that the C_α_ atoms of the residues H982 and H985 and the corresponding scissile phosphorus are 7.3 and 9.6 Å apart, respectively; meanwhile, N_δ_ atoms of their imidazole groups point toward the scissile phosphorus. Therefore, these two amino acids might be general bases. To identify the roles of these residues, through site-directed mutagenesis and cleavage activity detection, we first confirmed whether they are critical during the NTS hydrolysis. In our results, the mutant H982A is still able to cleave the target DNA and almost maintains the same cleavage activity as the wild-type SpCas9 (lanes 3 and 4 in Figure 3B). In contrast, the cleavage activity of the mutant H985A is significantly decreased (lane 6 in Figure 3B). Therefore, H985 is a key residue in the RuvC catalytic center and appears to play a more critical role than H982.

To further clarify the precise roles of the H982 and H985, we continued to examine the cleavage activities of several mutants, including H983A + H985A, H982A + H983A, H982A + H985A, H982A + H983D, H982A + H983A + H985A, and H982A + H983D + H985A (Figure 3B). Among them, the cleavage activity of the mutant H983A + H985A is still partly retained (lane 7 in Figure 3B). Therefore, H982 could be a general base to activate the water molecule for the nucleophilic attack of the phosphodiester bond. However, the cleavage activity of the mutant H982A + H983A is removed (lane 8 in Figure 3B). This seems to indicate that the H985 is not a Lewis base. But, it is because the distance between the H985 and the scissile phosphorus is too far in the mutant H982A + H983A that the H985-activated water molecule may not complete the nucleophilic attack of the scissile phosphorus (Supplementary Figure 9). This is also supported by site-directed mutagenesis because the lost activity of the mutant H982A + H983A is reversed by the mutant H982A + H983D (lane 15 in Figure 3B). Thus, H985 could be also a general base like H982.

Meanwhile, in the RuvC catalytic center, to rule out the possibility of independence on a general basis during the NTS hydrolysis, we evaluated the cleavage activities of the mutants H982A + H985A and H982A + H983D + H985A. These two mutants could not cleave the target DNA (lanes 9 and 10 in Figure 3B), similar to the mutant H982A + H983A + H985A (lane 16 in Figure 3B). Therefore, in the RuvC domain of SpCas9, the cleavage of the NTS is dependent on the general bases. Moreover, the chemical rescue by imidazole also supports this point (Supplementary Figure 10), because the lost activity of SpCas9 mutant H982A + H983D + H985A can be recovered by adding imidazole. Thus, the hydrolyzing of the NTS needs the residues of acting as the general bases besides the residues of stabilizing the Mg^2+^ ions. In the earlier studies, although the understanding of the RuvC domain has made significant advances, the residue H983 has always been regarded as a pseudo general base (Nishimasu et al., 2014; Jiang et al., 2016; Zuo and Liu, 2016; Palermo, 2019). However, the general bases could be residues of H982 and H985 from our studies. Taken together, the above results indicate that the H983 acts as a stabilizer of the Mg^2+^ ion, and reveal that both H982 and H985 serve as general bases to activate water molecules for the nucleophilic attack of the phosphodiester bond.

Based on the above results and the two-metal-ion mechanism (Steitz and Steitz, 1993; Yang, 2008), we propose a revised model for the RuvC catalytic center to hydrolyze the NTS (Figure 3C). First, two Mg^2+^ ions are jointly coordinated by the phosphate group of the NTS (between bases –3 and –4), water molecules, and four residues (D10, E762, H983, and D986). Among them, the Mg^2+^ ions A and B are about 4 Å apart, and the distances between Mg^2+^ ions and the corresponding ligands are about 2 Å. This coordination architecture facilitates the RuvC catalytic center and the phosphate backbone to get close to each other and then forms the reaction conformation for the nucleophilic attack on the scissile phosphodiester (reactant conformation in Figure 3C). Then, the imidazole groups of H982 and/or H985 capture the proton of the coordinated water and generate the nucleophile; meanwhile, two Mg^2+^ ions move toward each other to facilitate the activated water to attack the phosphodiester bond (pro-Rp oxygen). Subsequently, the nucleophile and the leaving group moieties are transiently bonded to the phosphorus for forming a transition state (transition state in Figure 3C). Finally, the nucleophile attacks the phosphorus (P5′) and the leaving groups depart simultaneously (product release in Figure 3C).

### SpCas9 Variants With Reduced Off-Target Effects

**FIGURE 4:**
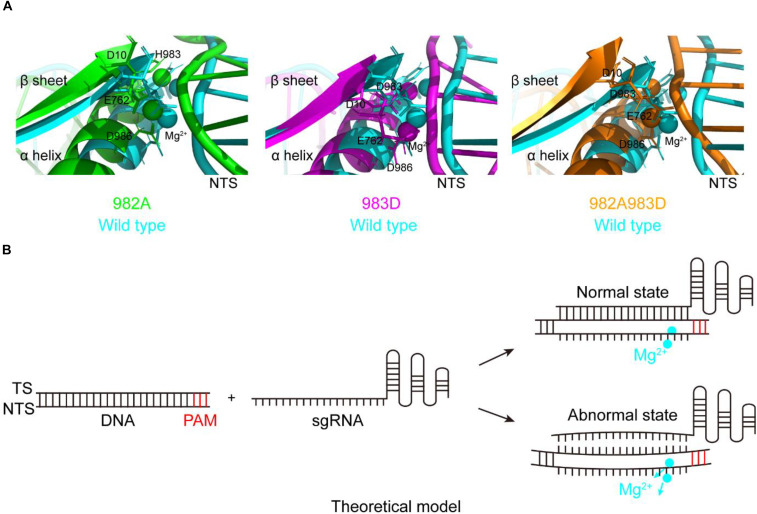
The movement of the non-target strands (NTS) in the PAM distal ends. **(A)** In the RuvC active centers of SpCas9 mutants, the NTSes of the PAM distal ends are shifted due to the comformational change of the RuvC catalytic centers (especially magnesium ions). **(B)** The model of reducing off-targets of SpCas9. In these mutants (H982A, H983D, and H982A + H983D), when sgRNAs are not matched with the NTS of the substrate DNA (an abnormal state), the DNA may be not cleaved by SpCas9 mutants.

During studying for the functions of the residues H982, H983, and H985, we have obtained three mutants with the DNA cleavage activity: H982A, H983D, and H982A + H983D. Interestingly, in these mutants, the MD simulations showed that the conformation changes of the RuvC catalytic centers remarkably cause a shift of the NTS that interacts with the Target strand (TS) of the PAM-distal end (Figure 4A), comparison of that of wild type; Also, stability shift assay showed that the stability of these mutants become high, especially H982A + H983D (Supplementary Figure 12). For this, we proposed a model: in the active center where the conformation of the key sites was changed, when sgRNA combined to DNA, if sgRNA completely matched with NTS in DNA, the DNA may be cleaved by SpCas9 variants; on the contrary, the DNA may not be sheared by SpCas9 variants (Figure 4B). Therefore, we speculated that these mutants may hinder complete hybridization between the sgRNA and the DNA to improve their specificity when there are no highly matched Watson–Crick base pairs between the sgRNA and the TS.

**FIGURE 5:**
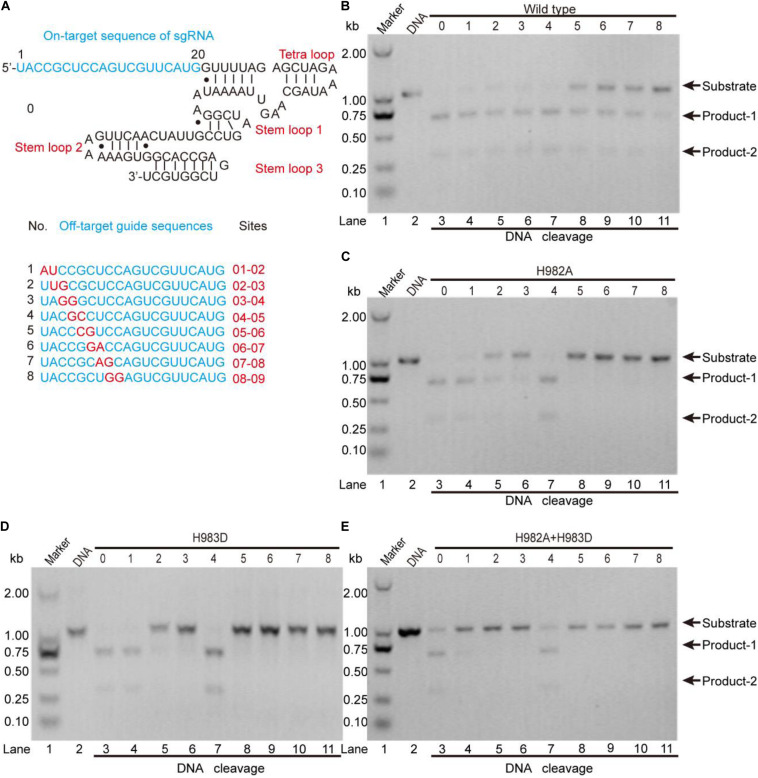
The detection of the *in vitro* off-target effects. **(A)** The sequences of the sgRNA and its mutants. The on-target sequence of the sgRNA shows in No. 0; the off-target sequences show in Nos. 1, 2, 3, 4, 5, 6, 7, and 8. **(B–E)** The detection of the off-target effects for the wild-type SpCas9 and its mutants. The target DNA molecules are cleaved into two products (product-1 and -2).

To clarify this, we detected the off-target effects of the mentioned mutants by agarose gel electrophoresis (Figure 5). As seen, for the wild-type, all sgRNAs can guide the DNA cleavage (Figure 5B), indicating that the off-target effects of the wild-type SpCas9 are very severe. For the mutants H982A and H983D, only sgRNAs 1, 2, 3, and 4 can guide them to cleavage the target DNA (Figures 5C,D), demonstrating that the mutants H982A and H983D could reduce the off-target effects. For the mutant H982A + H983D, only sgRNAs 1 and 4 are capable of guiding the DNA cleavage (Figure 5E), illustrating that this mutant greatly decreases the off-target effects. In addition, to evaluate the kinetics of these mutants, we also performed the time-course cleavage reaction, and qualitatively observed that their reaction rates had the following trend: SpCas9 > H982A > H983D ≈ H982A + H983D (Supplementary Figure 13). In general, in the three mutants, the mutant H982A + H983D may have more potential application value. Despite it being necessary to conduct *in vivo* studies for these mutants, it is not the focus of the thesis; thus, *in vivo* studies related to these mutants will be exhibited in future work.

In the previous studies, the strategies to decrease the SpCas9 off-target effects mainly include the following several aspects: (1) using a pair of catalytic-inactive SpCas9 nucleases which fused to a *Fok*I nuclease domain (Tsai et al., 2014); (2) truncating the guide sequence of the sgRNA at 5′ end (Fu et al., 2014); (3) decreasing the number of the active SpCas9 in the cell (Hsu et al., 2013); (4) using a pair of SpCas9 nickase mutants to produce double nicks for DNA (Ran et al., 2013); and (5) neutralizing positively charged residues within the NTS groove (Slaymaker et al., 2016). Nevertheless, few studies focused on the relationship between the specificity of SpCas9 and the residue types of its catalytic center. In our results, we found that the SpCas9 mutants may possess high catalytic specificity by changing the catalytic residues of the RuvC catalytic center.

## Conclusion

In summary, we refined the cryo-EM structure of the SpCas9–sgRNA–DNA complex in the DNA cleavage state. This active structure presents an atomic model for the HNH and RuvC active-sites in complex with DNA, Mg^2+^ ions, and water molecules, and thereby identifying the catalytic roles of the residues D10, E762, H982, H983, H985, and D986 in the RuvC active sites. Moreover, our catalytic model led to a new engineered SpCas9 variant (SpCas9-H982A + H983D) with reduced off-target effects. Hence, this study not only provides new mechanistic insights into the DNA cleavage by CRISPR-Cas9 but also offers useful guidance for engineering new CRISPR-Cas9 systems with improved specificity.

## Materials and Methods

### Expression and Purification of Cas9 Proteins

The sequences of encoding SpCas9 and its mutants (primers in Supplementary Table 1, mutants in Supplementary Table 2) were inserted between the restriction sites (*Nde*I and *Xho*I) of the plasmid pET-21, and their N-terminals were fused with a His × 6 tag and Tobacco etch virus (TEV) protease cleavage site (Huai et al., 2017). The integrative plasmid was transformed into Rosetta (DE3) competent cells (TANGEN), which were cultivated in Terrific Broth (Sangon Biotch, Shanghai, China) containing antibiotics (100 μg/ml Amp and 30 μg/ml Cm) at 37°C on a 200 rpm shaker. When the absorption value (OD600) of the cell concentration in the bacteria solution was approximately 0.8–1.0 (Huai et al., 2017), a.5 mM-IPTG was added to the bacteria solution to induce protein expression at 16°C on a 160 rpm shaker for approximately 20 h. Next, harvest cells were collected by centrifugation at 4°C and 5,000 rpm for 10 min and were lysed by sonication (power output 5, pulse-on 3 s, pulse-off 3 s, for a total of 10 min) to release the protein in the lysis buffer (20 mM HEPES, 500 mM KCl, pH = 7.5, 0.2 μm filtered and degassed) (Huai et al., 2017). Then, the protein was bound to Ni-NTA agarose beads (Qiagen, Shanghai, China) in the ice bath on a 150 rpm shaker for at least 1.5 h, was washed by the wash buffer (20 mM HEPES, 500 mM KCl, 1% sucrose, pH = 7.5, 0.2 μm filtered and degassed) at 2 ml/min, and eluted by the eluent buffer (wash buffer with 20, 30, 50, 100, and 250 mM imidazole, respectively; pH = 7.5,0.2 μm filtered and degassed) at 2 ml/min (Anders et al., 2014; Huai et al., 2017). Subsequently, the protein was incubated with TEV protease overnight at 4°C to remove the 6× His tag. Finally, the protein was further purified by SP Sepharose HiTrap column (elution with a linear gradient of 0.1–1 M KCl) and Superdex 200 16/60 column from GE Life Sciences (elution with 20 mM HEPES, 500 mM KCl, pH = 7.5) (Anders et al., 2014; Jiang et al., 2016; Huai et al., 2017), and was concentrated by 100 kDa MWCO centrifugal filter (Merck Millipore) to store in the storage buffer (20 mM HEPES, 150 mM KCl, 1 mM DTT, glycerol 50%, pH = 7.5) (Huai et al., 2017). All proteins were detected by SDS-PAGE (SDS-PAGE image of part proteins are shown in Supplementary Figure 11).

Note: The base sequences (Supplementary Table 3) of SpCas9 were mutated using the Muta-diret^TM^ site-Directed Mutagenesls Kit (Beijing SBS Genetech Co, Ltd., Beijing, China^1^, cat.no.SDM-15) to obtain every mutant (Supplementary Table 2) in the present text. Reaction system (50 μl) includes 10 × reaction buffer (5 μl), sample plasmid (10 ng/μl, 2μl), primer F/R (10 pmol/μl, 1μl), dNTP mixture (each 2.5 mM, 2 μl), ddH2O (38 μl), Muta-direct^TM^ enzyme (1 μl). A reaction is ended under the conditions (95°C 30 s, 55°C 30 s, 72°C 10 min, 15–18 cycles), Mutazyme^TM^ (10 U/μl, 1 μl) was added to the reaction system to digest methylated plasmids; Then, mutant plasmids in the reaction system were transformed into competent cells.

### Preparation of Nucleic Acids

A 98-nt sgRNA was *in vitro* transcribed and purified using the MEGAshortscript T7 Transcription Kit and the MEGAclear Transcription Clean-Up Kit from Thermo Fisher Scientific (China) Co., Ltd., Pudong New Area, Shanghai, China. A 920-bp target DNA contained PAM (TGG) was amplified by the Ultra HiFidelity PCR Kit (TIANGEN, Sichuan, China) at the following conditions (94°C 30 s, 55°C 30 s, 72°C 1.5 min, 30 cycles), and was purified using the AxyPrep^TM^ DNA Gel Extraction Kit (Axygen Biotechnology, Taizhou, China). The purified sgRNA and DNA were resuspended using the desired solution and volume, and stored at −80°C.

### Activity Detection of Cas9 Proteins

Methods of activity can be detected in three ways: (1) *In vitro* activity assays: the mole numbers (200 nM, 1 μl) of Cas9 proteins (Supplementary Table 2) are equal to that of sgRNA, and both cas9 protein and sgRNA were mixed and incubated in the buffer (20 mM HEPES, 100 mM KCl, 1 mM DTT, 0.5 mM EDTA, 2 mM MgCl2, 5% glycerol, pH = 7.5) at 37°C for 30 min; the buffer was added with 100–150 ng target DNA, and incubated another 1.5 h at 37°; the cleavage products were detected by gel electrophoresis on 1% agarose gel stained with 1 × GeneGreen nucleic acid dye (TIANGEN). (2) Chemical rescue of His982 and His985 by imidazole: this process was similar to (1), with the only difference being the addition of a step: “the solution was mixed with 2 μl imidazole (500 mM) and incubated overnight at 37°C” before the detection of products. (3) *In vitro* detection of the off-target effects: the process was similar to (1), with the difference being sgRNA (continuous mutation with two bases as a unit in 20-nt sequence for target DNA) and the incubation time (4 h) after adding target DNA.

### Building and Refinement of Atomic Models

The atomic model of the ternary complex (SpCas9–sgRNA–DNA) was built according to the cryo-EM density maps (end-21308.map and end-0584.map) (Zhu et al., 2019; Lapinaite et al., 2020), and the previous conformation of SpCas9 (PDB IDs: 5Y36, 6O0Y, and 6VPC) (Huai et al., 2017; Zhu et al., 2019; Lapinaite et al., 2020). First, the initial model of the all-atom ternary complex was constructed through the homology modeling method MODELLER using the aforementioned PDBs as templates (Fiser and Sali, 2003; Huai et al., 2017; Zhu et al., 2019; Lapinaite et al., 2020). Next, the initial position of the HNH domain was modified based on reference structures (PDB ID 6O0Y) and its cryo-EM density (end-0584.map) (Zhu et al., 2019); at the same time, the RuvC domain was amended using 6VPC and its cryo-EM density (end-21308.map) as references. Then, the position of the Mg^2+^ ions in the active sites was confirmed by NAMD with a 12–6–4-type multisite model to obtain the experimental coordination patterns (Li and Merz, 2014; Liao et al., 2017). Finally, the initial all-atom model of the ternary complex was automatically refined by Situs-3.1, Rosetta macromolecular modeling suite, and MD simulations (Wriggers, 2010; Leaver-Fay et al., 2011; Mirjalili et al., 2014).

### Molecular Dynamics Simulations

The MD simulations with explicit water models were conducted using NAMD (Phillips et al., 2005). The CHARMM36 force field (Hart et al., 2012) and the TIP3P water (Jorgensen et al., 1983) model were employed to model the simulation system of the ternary complex with the water solvent. To build a simulation system, some simulation parameters were adjusted: the all-atom structure of the ternary complex was solvated in the center of a cubic water box with a minimum distance of 12 Å from the complex surface to the edge of the box; the Na^+^ and Cl^–^ ions were used to mimic an ionic concentration of.15 M in the system, including the certain number of additional Na^+^ ions that neutralize the net negative charge of the complex. To conduct the simulations, periodic boundary conditions were used; the van der Waals interactions were treated with a cut-off distance of 10 Å using a smooth switching function from 8 Å; the electrostatic interactions were calculated with particle mesh Ewald (PME) method using a local interaction distance of 10 Å; the SHAKE algorithm was employed to constrain bonds involving hydrogen atom, and thereby a time step of 2.0 fs was used. Ultimately, the simulations were performed in the isobaric-isothermal (NPT) ensemble, at a constant pressure of 1 bar and a constant temperature of 298 K controlled by Langevin dynamics.

## Data Availability Statement

The datasets presented in this study can be found in online repositories. The names of the repository/repositories and accession number(s) can be found in the article/Supplementary Material.

## Author Contributions

QH supervised the project. HT, HY, WD, GL, and DX expressed and purified proteins and conducted the DNA cleavage assay. HT and QH carried out the building, refinement of atomic models, and wrote the manuscript. All authors contributed to the article and approved the submitted version.

## Conflict of Interest

The authors declare that the research was conducted in the absence of any commercial or financial relationships that could be construed as a potential conflict of interest.

## Footnotes

1 http://www.sbsbio.com
