# Erratum: Active-Site Models of *Streptococcus pyogenes* Cas9 in DNA Cleavage State

**DOI:** 10.3389/fmolb.2021.719327

**Published:** 2021-06-18

**Authors:** Frontiers Production Office

**Affiliations:** Frontiers Media SA, Lausanne, Switzerland

**Keywords:** Gene editing, CRISPR-Cas9, DNA cleavage mechanism, Molecular dynamics, off-target effects

Due to a production error, the second sentence in the abstract was replaced with the definition of HNH. A correction has been made to the section.

**Abstract**:

“CRISPR-Cas9 is a powerful tool for target genome editing in living cells. Significant advances have been made to understand how this system cleaves target DNA. However, due to difficulty in determining active CRISPR-Cas9 structure in DNA cleavage state by X-ray and cryo-EM, it remains uncertain how the HNH and RuvC nuclease domains in CRISPR-Cas9 split the DNA phosphodiester bonds with metal ions and water molecules. Therefore, based on one- and two-metal-ion mechanisms, homology modeling and molecular dynamics simulation (MD) are suitable tools for building an atomic model of Cas9 in the DNA cleavage state. Here, by modeling and MD, we presented an atomic model of SpCas9-sgRNA-DNA complex with the cleavage state. This model shows that the HNH and RuvC conformations resemble their DNA cleavage state where the active-sites in the complex coordinate with DNA, Mg2+ ions and water. Among them, residues D10, E762, H983 and D986 locate at the first shell of the RuvC active-site and interact with the ions directly, residues H982 or/and H985 are general (Lewis) bases, and the coordinated water is located at the positions for nucleophilic attack of the scissile phosphate. Meanwhile, this catalytic model led us to engineer new SpCas9 variant (SpCas9-H982A + H983D) with reduced off-target effects. Thus, our study provides new mechanistic insights into the CRISPR-Cas9 system in the DNA cleavage state, and offers useful guidance for engineering new CRISPR-Cas9 editing systems with improved specificity.”

The authors apologize for this error and state that this does not change the scientific conclusions of the article in any way. The original article has been updated.

