# Supplementary material for: Active-Site Models of Streptococcus pyogenes Cas9 in DNA Cleavage State
Source: Front Mol Biosci. 2021 Apr 21;8:653262. doi: 10.3389/fmolb.2021.653262 (PMC8112549; doi:10.3389/fmolb.2021.653262)
Supplement: Supplementary file 1 [file Data_Sheet_1.pdf]

Supplementary Material

Active-site models of *Streptococcus pyogenes* Cas9 in DNA cleavage state

Honghai Tang[1], Hui Yuan[1], Wenhao Du[1], Gan Li[1], Dongmei Xue[1], & Qiang Huang[1,2]*

[1]*State Key Laboratory of Genetic Engineering, MOE Engineering Research Centre of Gene Technology, School of Life Sciences, Fudan University, Shanghai, 200438, China.* [2]*Multiscale Research Institute of Complex Systems, Fudan University, Shanghai 201203, China.*

*Correspondence and requests for materials should be addressed to Q.H. (Email: huangqiang@fudan.edu.cn).

Contents

Figures

Fig. S1. The structures of SpCas9……………………….….………………………………....S1

Fig. S2. The structural alignments of SpCas9 without nucleic acids……….…….………....S2

Fig. S3. Rigid-body fitting of atomic model into cryo-EM density…………………….…......S3

Fig. S4. The locations of key sites in the catalytic centres……………………………….….........S4

Fig. S5. The relative position between RuvC key sites and magnesian ions……….….........S5

Fig. S6. The stable state of the tenary complex in catalytic cleavage……………………....S6

Fig. S7. Comparison of the catalytic centres from different proteins……………………….....S7

Fig. S8. The distances between Mg^{2+} ions A and B…………….…………….……………....S8

Fig. S9. The distances between the H985 and the scissile P atoms……………………….....S9

Fig. S10. Chemical rescue of the SpCas9 mutants by imidazole………………….…...…..S10

Fig.S11. The purification of the SpCas9 mutants……………….………..…...........S11

Fig.S12. The stability evaluation of SpCas9 mutants……………….………..…........S12

Fig. S13. The time-course cleavage activity of SpCas9 mutants.…….……….….…..........S13

Fig. S14. Cryo-EM micrographs of SpCas9 mutants…………...……….….....................S14

Tables

Table S1. Primers of the SpCas9 mutants…......……….……….……………………....S15

Table S2. The SpCas9 mutants………………………….....………..…………………..S16

Table S3. The base sequences of SpCas9…………………………….....………………...S17

Figures

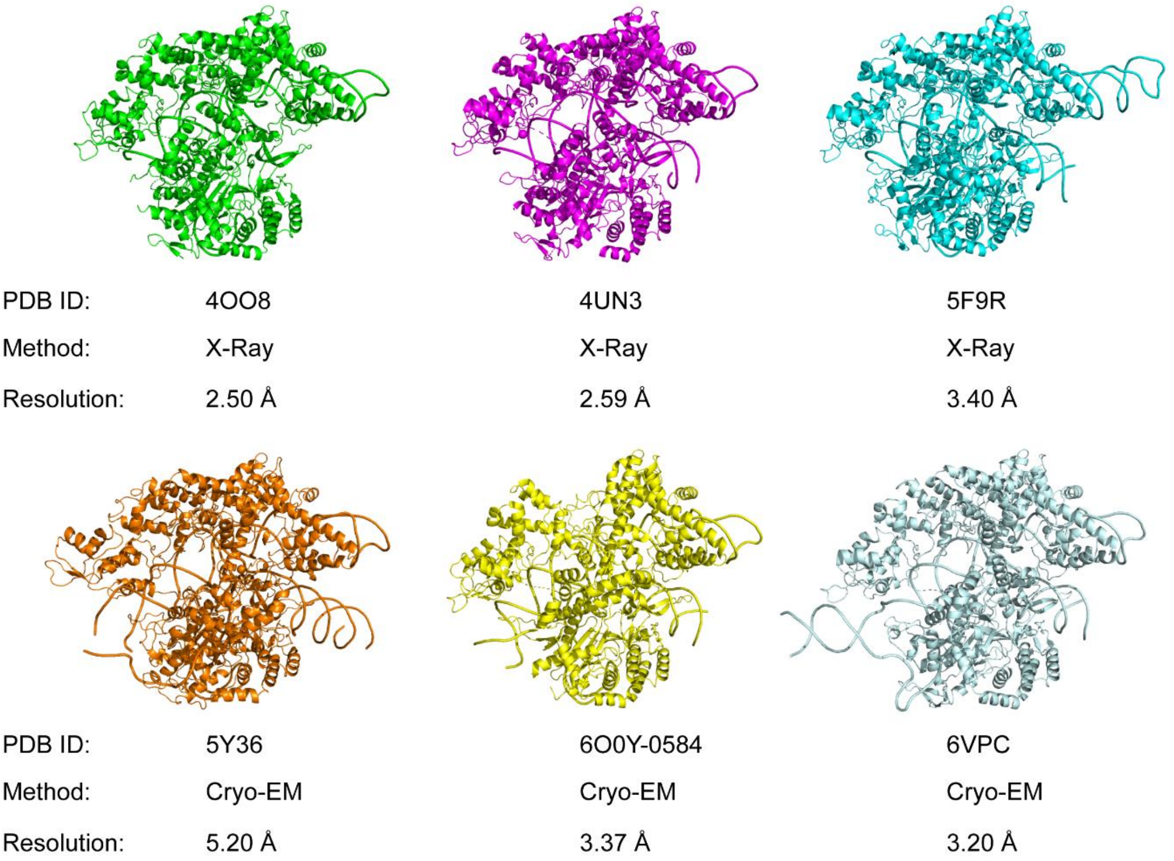

Fig. S1. The structures of SpCas9. All atom models 4OO8, 4UN3, 5F9R, 5Y36, 6O0Y, and 6VPC are SpCas9-sgRNA-DNA ternary complexes.

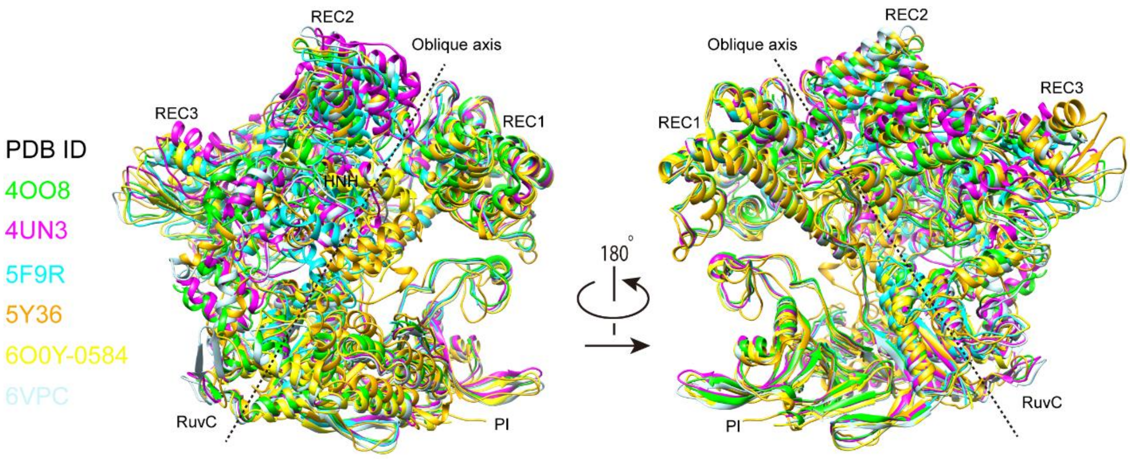

Fig. S2. The structural alignments of SpCas9 without nucleic acids. These structures come from 4OO8, 4UN3, 5F9R, 5Y36, 6O0Y, and 6VPC, respectively. The domains below the oblique axis include REC1 and PI, whereas the domains above the oblique axis contain REC2, REC3, HNH, and RuvC.

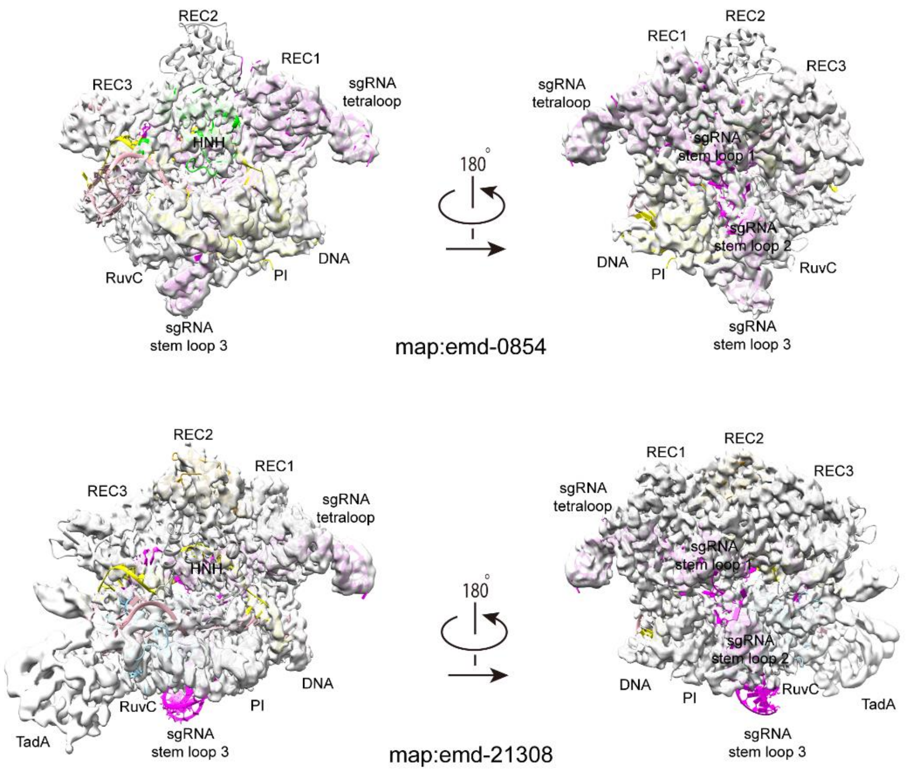

Fig. S3. Rigid-body fitting of atomic model into cryo-EM density. The atomic model of SpCas9 superposes into the 3.37-Å and the 3.20-Å cryo-EM density maps at a lower contour level (0.009), respectively.

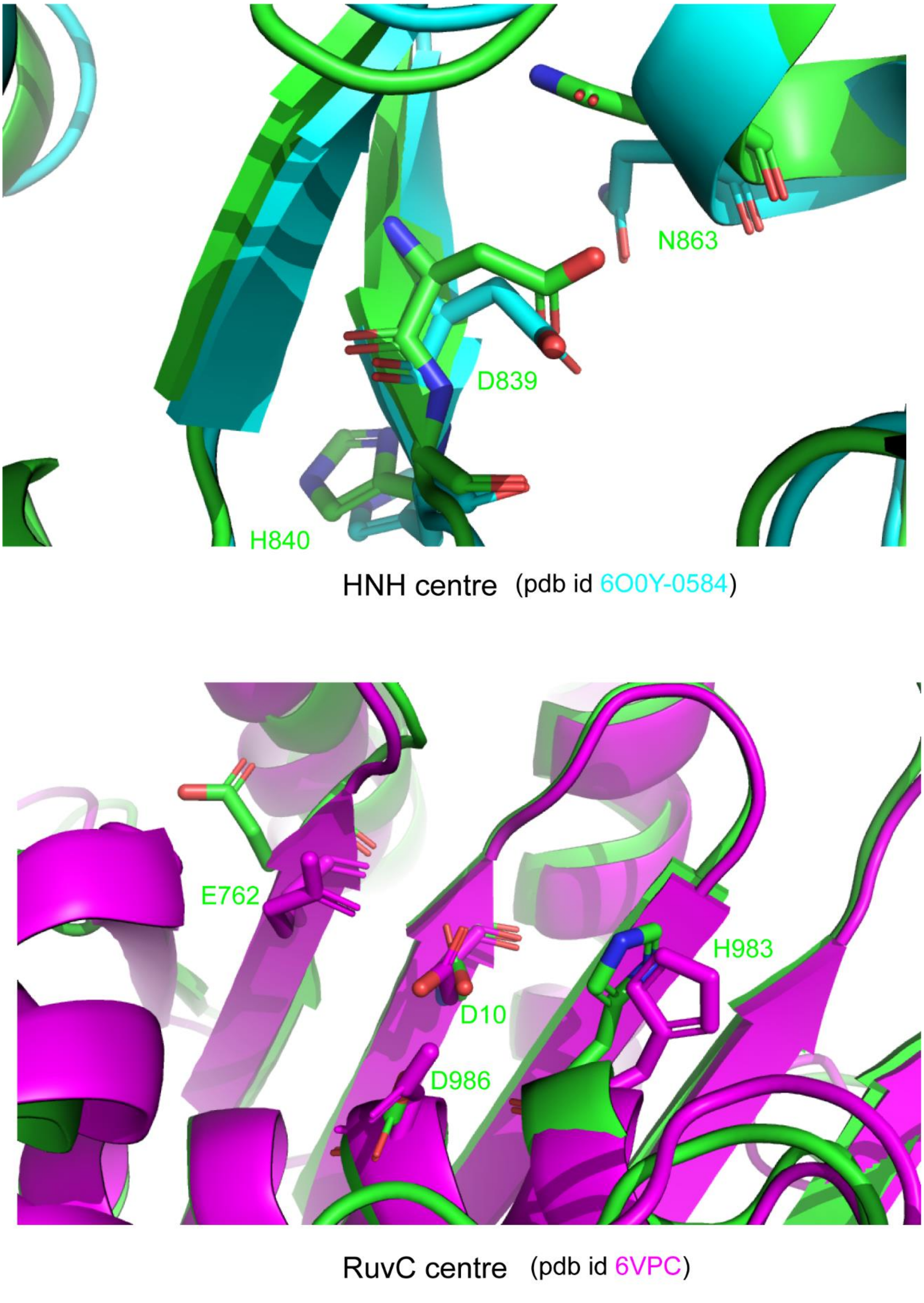

Fig. S4. The locations of key sites in the catalytic centres. The critical residues of HNH centre are D839, H840, and N863; the key residues of RuvC centre are D10, E762, H983, and D986.

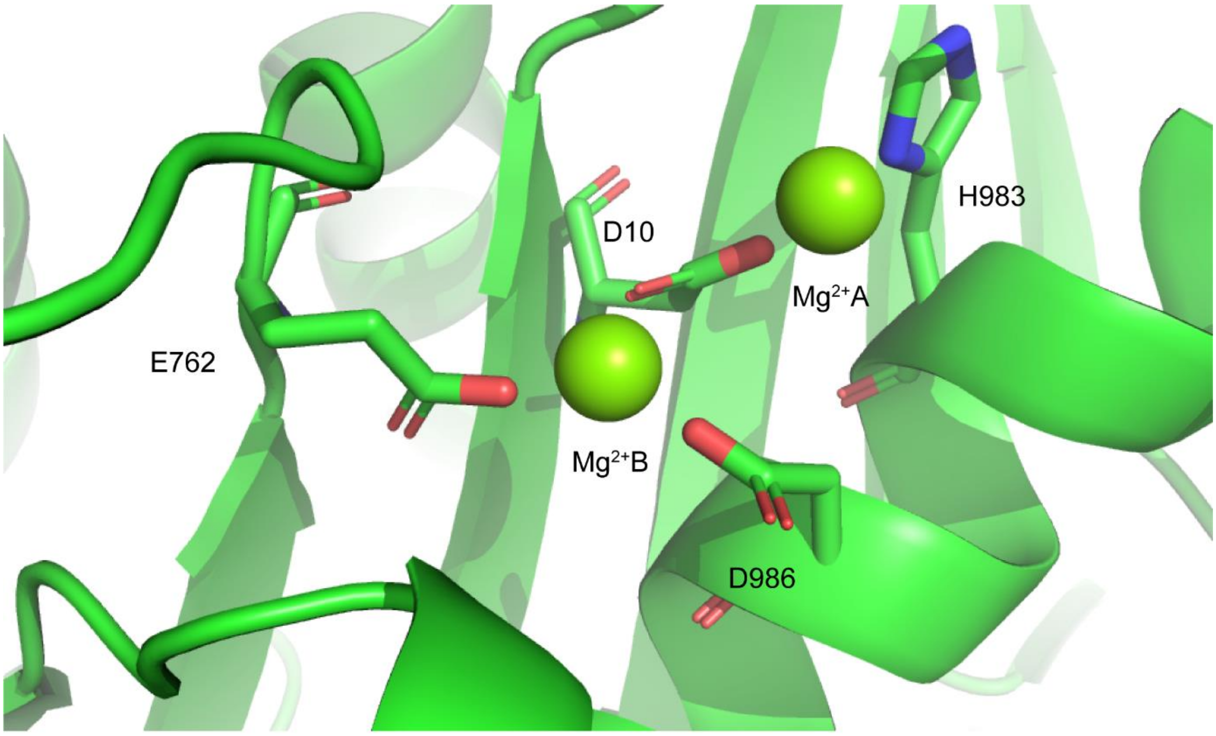

Fig. S5. The relative position between RuvC key sites and magnesian ions. Key sites and magnesian ions are in the active centre of the RuvC domain.

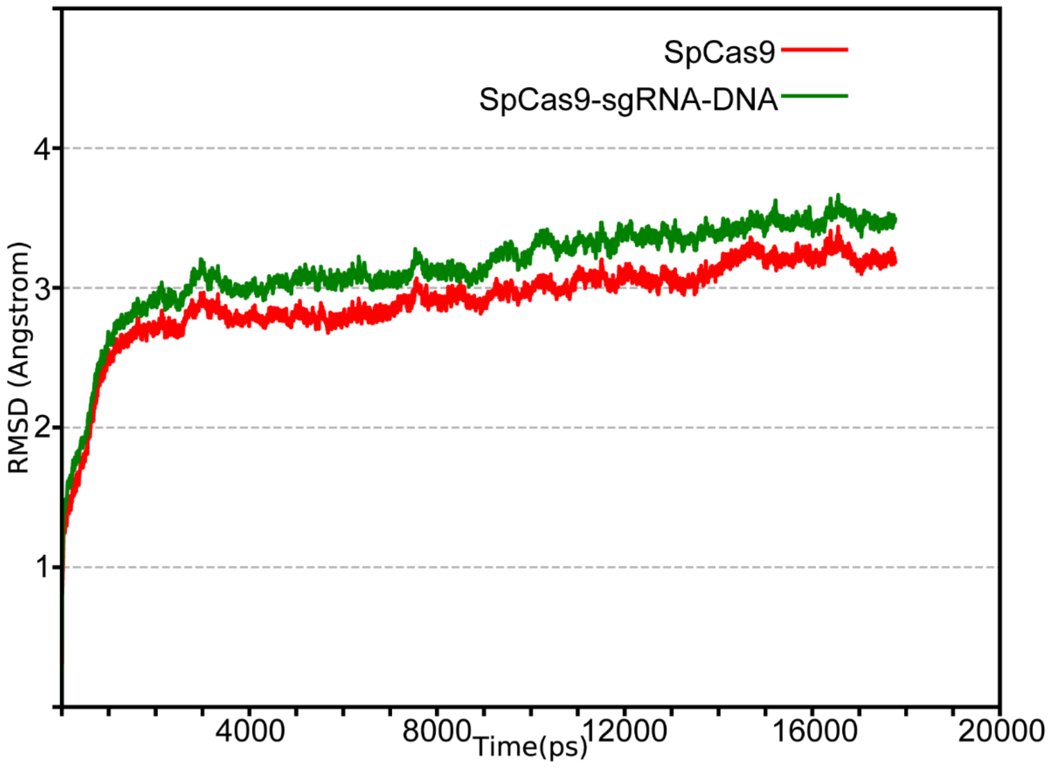

Fig. S6. The stable state of the tenary complex in catalytic cleavage. The RMSDs of SpCas9 monomer and SpCas9-sgRNA-DNA ternary complex keep around 3 angstrom during DNA catalytic hydrolysis.

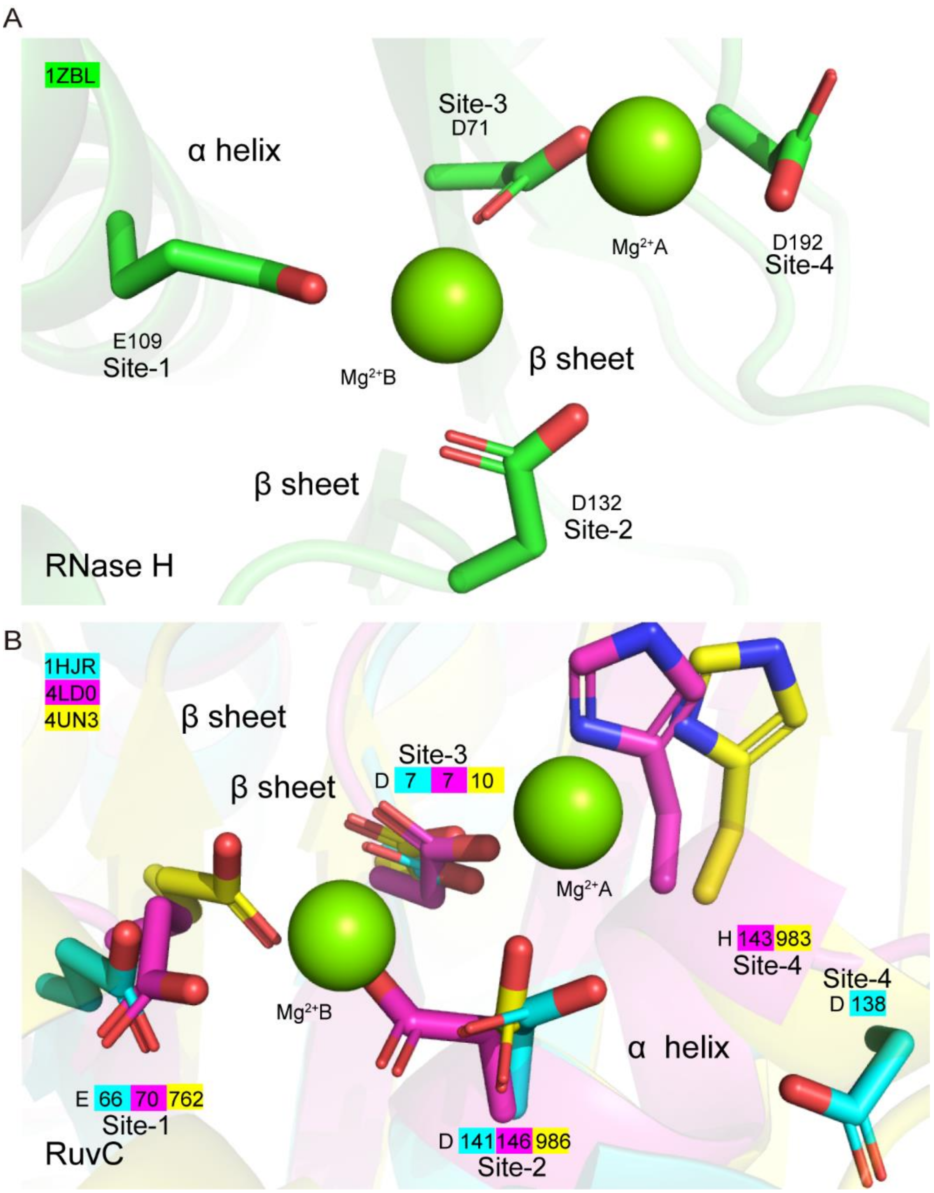

Fig. S7. Comparison of the catalytic centres from different proteins. (A) The active-site conformation of the catalytic centre in the RNase H (PDB ID: 1ZBL). (B) The active-site conformations of the catalytic centres of the RuvCs (PDB IDs: 1HJR, 4LD0 and 4UN3, respectively).

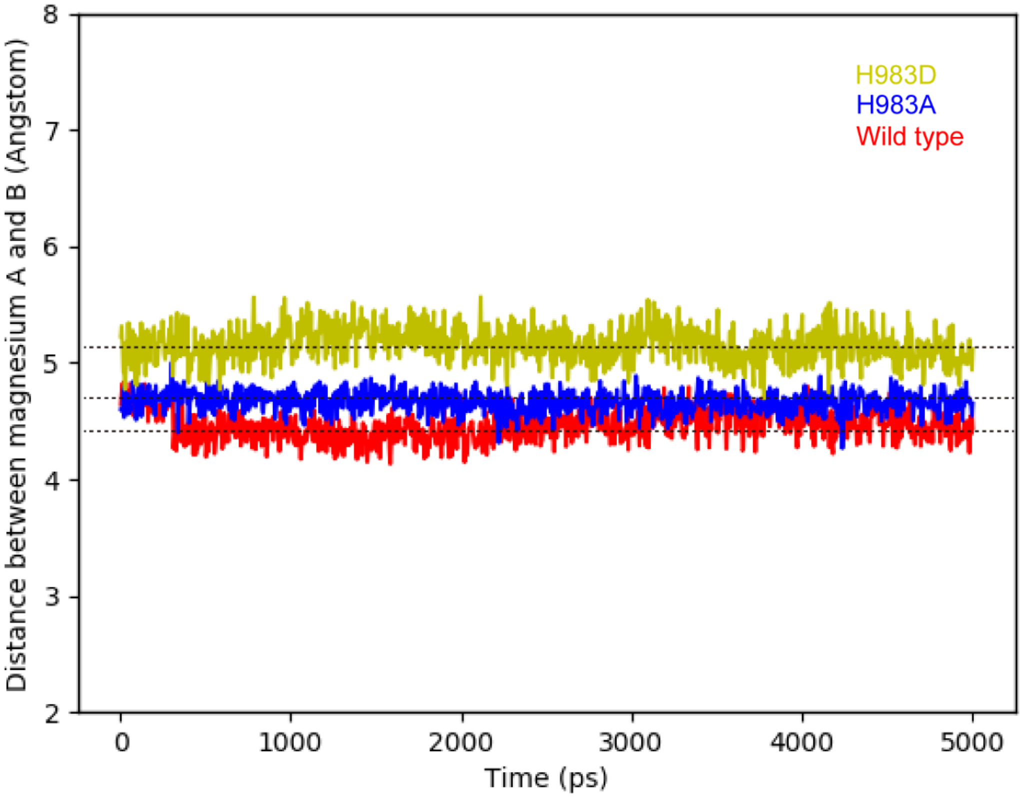

Fig. S8. The distances between Mg^{2+} ions A and B. The distances between the Mg^{2+} ions A and B in the wild-type SpCas9 and its mutants (H983A and H983D) were analysed by the molecular dynamics simulation, respectively.

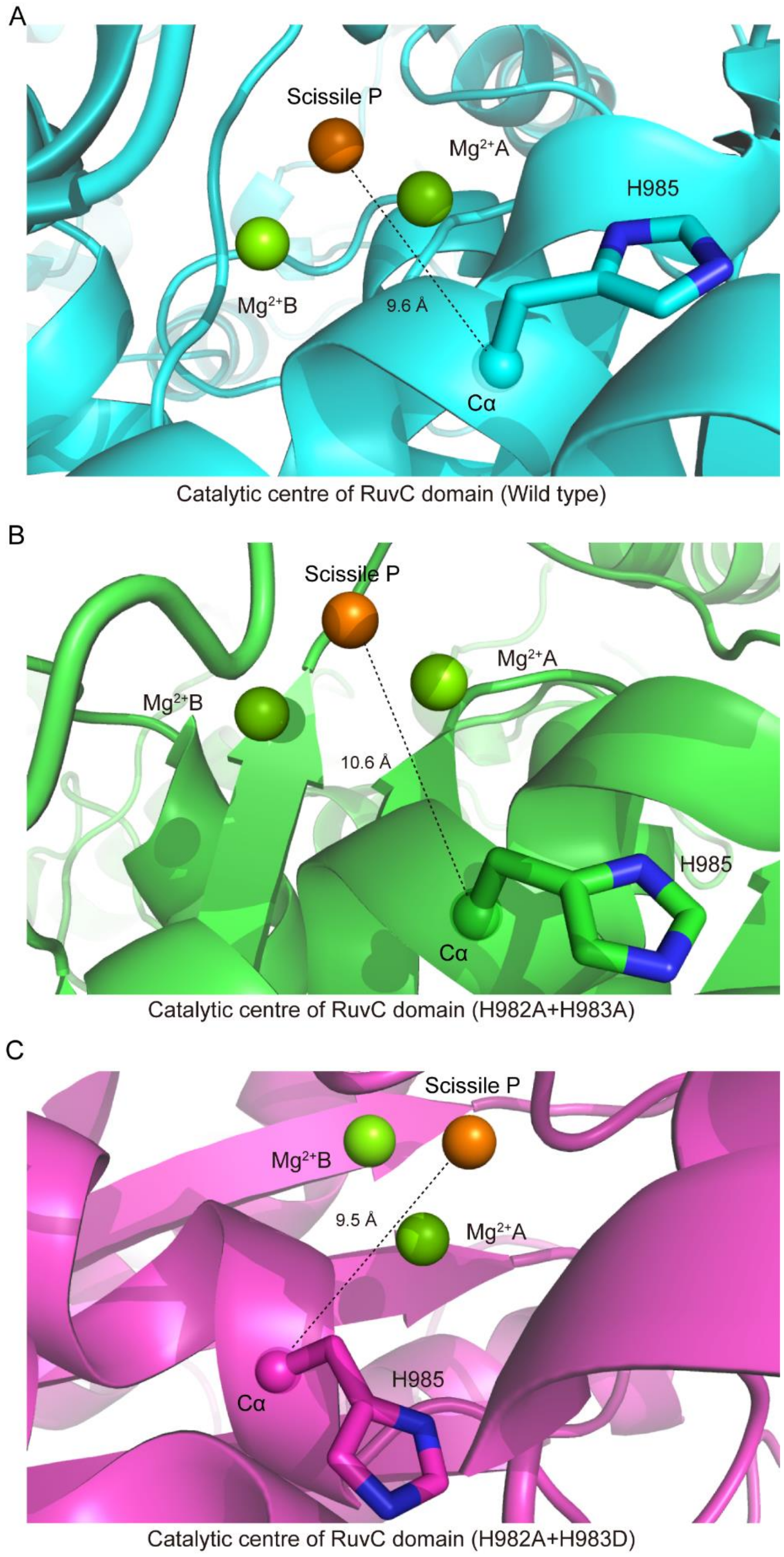

Fig. S9. The distances between H985 and scissile P atoms. (A) The distance from the scissile P atom to the Cα atom of the H985 in the wild-type SpCas9. (B, C) The distances from the scissile P atoms to the Cα atoms of the H985 in the SpCas9 mutants (H982A+H983A and H982A+H983D, respectively).

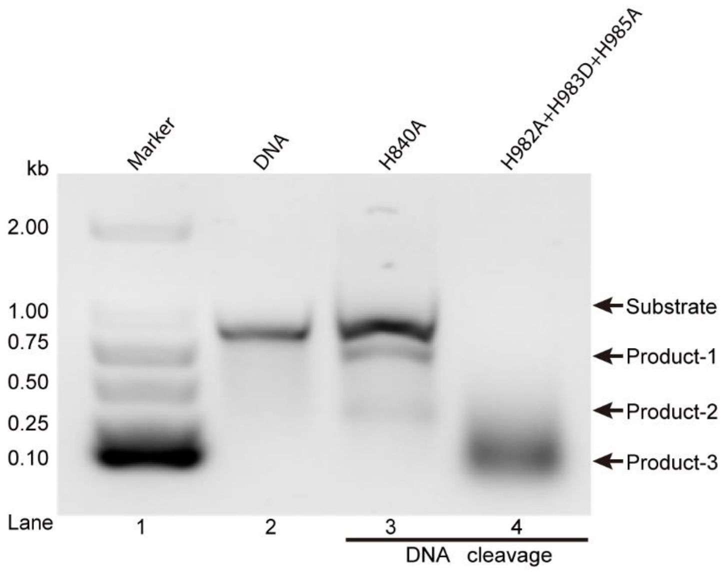

Fig. S10. The chemical rescue of the SpCas9 mutants by imidazole. The activities of the SpCas9 mutants (H840A and H982A+H983D+H985A) were recovered by chemical rescue of the imidazole.

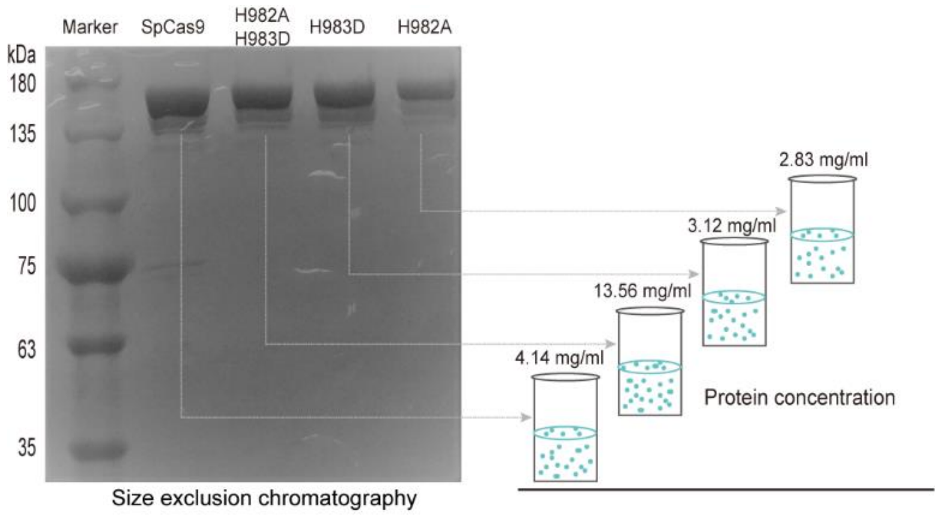

Fig. S11. The purification of the SpCas9 mutants. The SpCas9 mutants were purified by the size exclusion chromatography, and were condensed using 100 kDa MWCO centrifugal filter (Merck Millipore).

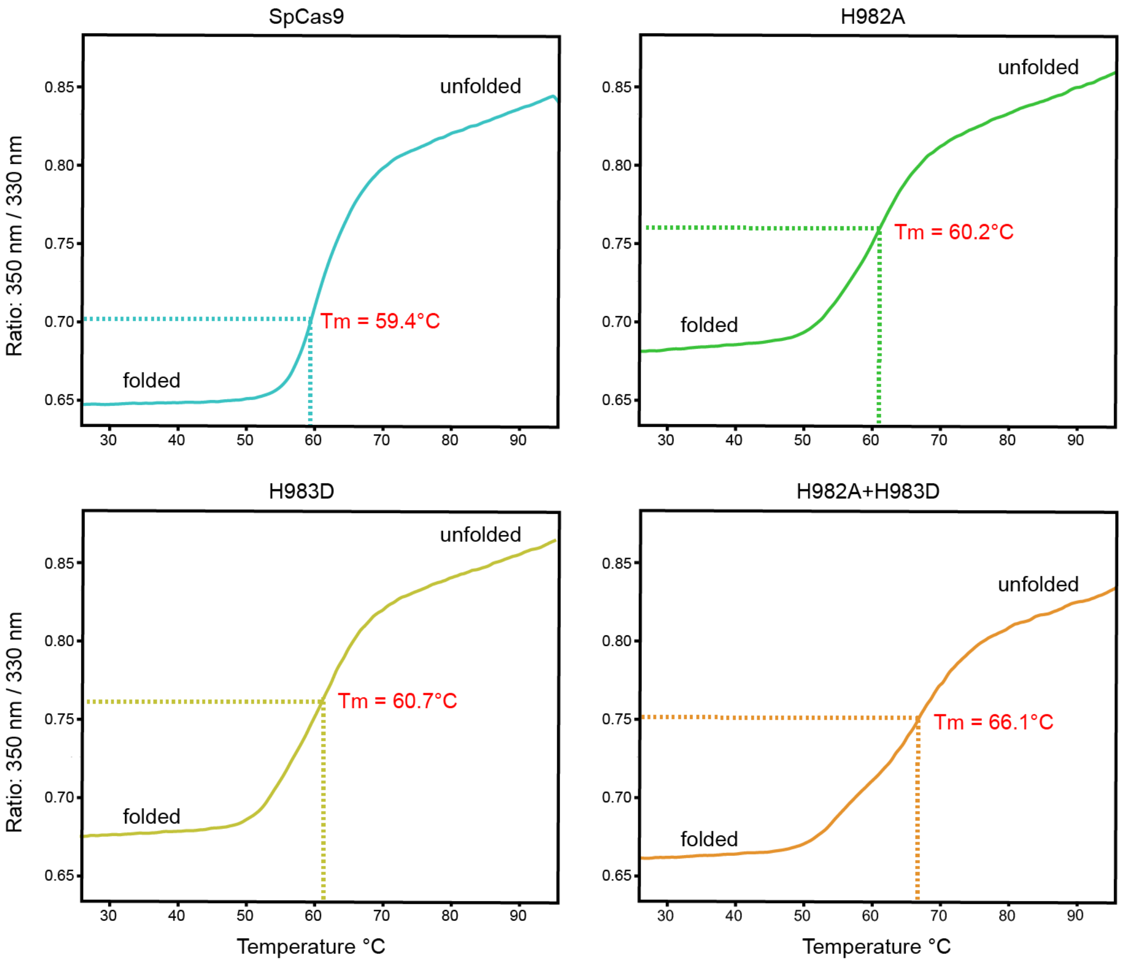

Fig. S12. The stability evaluation of SpCas9 mutants. The stability of SpCas9 mutants were analyzed by Nano Temper PR Panta (https://nanotempertech.com/prometheus). Onset temperatures of SpCas9, H982A, H983D, and H982A+H983D were 52.5, 46.8, 47.2, 42.3 ℃, respectively. T_m values of SpCas9, H982A, H983D, and H982A+H983D were 59.4, 60.2, 60.7, 66.1℃, respectively. Parameters including mass concentration (0.89 ml/ml), temperature rate (1 ℃/min), instrument precision (0.4). 330 nm and 350 nm represent wavelength of emitted light. Note that T_m difference between samples > 0.4 indicates their stabilities are different.

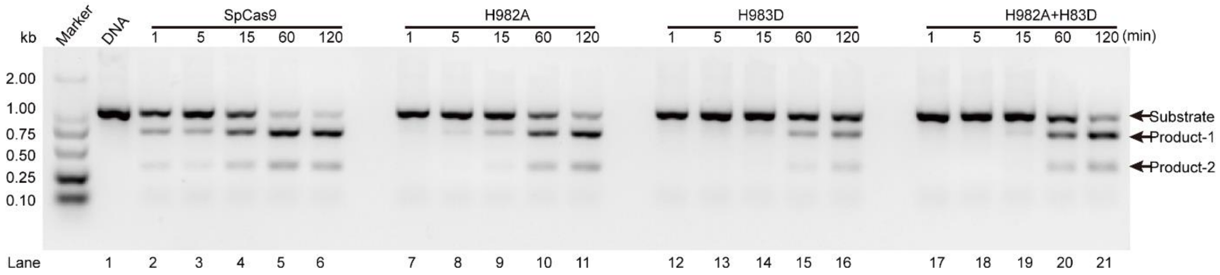

Fig. S13. The time-course cleavage activity of SpCas9 and its mutants. SpCas9 or its mutant was co-incubated with sgRNA and substrate DNA in the buffer (20 mM HEPES, 100 mM KCl, 1 mM DTT, 0.5 mM EDTA, 2 mM MgCl2, 5% glycerol, pH = 7.5) at 37℃ for 1, 5, 15, 60, 120 min, respectively. After protein denaturing at 70℃, the cleavage products were detected by gel electrophoresis on 1% agarose gel stained with 1 × GeneGreen nucleic acid dye (TIANGEN).

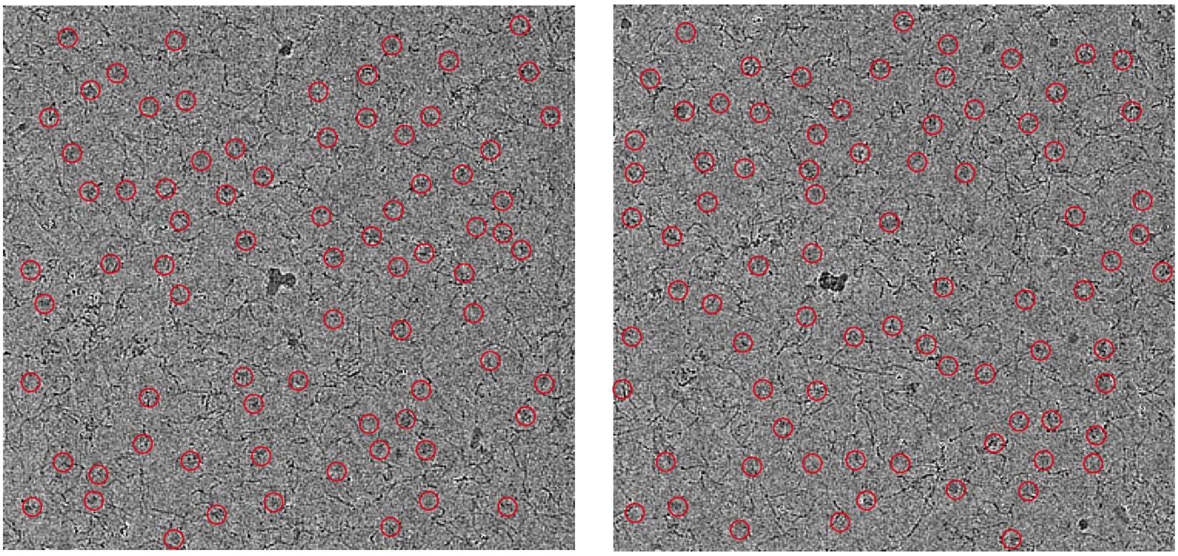

Fig. S14. EM micrographs of the SpCas9 mutants in the vitreous ice imaged by 300 kV cryo-EM by using similar experimental methods and the same Titan Krios microscope described in our previous study (Huai et al., 2017). The mutant particles in the micrographs are similar to those in Huai et al. (2017), e.g., as shown in its supplementary Fig. 3a, and therefore indicate that the mutants are in the monomer form.

Tables

Table S1. Primers of the SpCas9 mutants

No.	Primers	Sequences (5'→3')
01	E762A-F	GAGAATATCGTTATCGCGATGGCCCGAGAGAAC
02	E762A-R	GTTCTCTCGGGCCATCGCGATAACGATATTCTC
03	D839A-F	TCCGACTACGACGTGGCTCATATCGTGCCCCAG
04	D839A-R	CTGGGGCACGATATGAGCCACGTCGTAGTCGGA
05	H840A-F	CGACTACGACGTGGATGCTATCGTGCCCCAGTC
06	H840A-R	GACTGGGGCACGATAGCATCCACGTCGTAGTCG
07	N863A-F	ACAAGATCCGATAAAGCGAGAGGGAAGAGTGAT
08	N863A-R	ATCACTCTTCCCTCTCGCTTTATCGGATCTTGT
09	H983D-F	GATCAACAATTACCACGATGCGCATGATGCCTAC
10	H983D-R	GTAGGCATCATGCGCATCGTGGTAATTGTTGATC
11	H983A-F	GATCAACAATTACCACGCTGCGCATGATGCCTAC
12	H983A-R	GTAGGCATCATGCGCAGCGTGGTAATTGTTGATC
13	H982A-F	GAGATCAACAATTACGCCCATGCGCATGATGC
14	H982A-R	GCATCATGCGCATGGGCGTAATTGTTGATCTC
15	H985A-F	AATTACCACCATGCGGCTGATGCCTACCTGAAT
16	H985A-R	ATTCAGGTAGGCATCAGCCGCATGGTGGTAATT
17	H983A+H985A-F	AATTACCACGCTGCGGCTGATGCCTACCTGAAT
18	H983A+H985A-R	ATTCAGGTAGGCATCAGCCGCAGCGTGGTAATT
19	H982A+H983A-F	GATCAACAATTACGCCGCTGCGCATGATGCCTAC
20	H982A+H983A-R	GTAGGCATCATGCGCAGCGGCGTAATTGTTGATC
21	H982A+H983D-F	GATCAACAATTACGCCGATGCGCATGATGCCTAC
22	H982A+H983D-R	GTAGGCATCATGCGCATCGGCGTAATTGTTGATC
23	H982A+H985A-F	GATCAACAATTACGCCCATGCGGCTGATGCCTAC
24	H982A+H985A-R	GTAGGCATCAGCCGCATGGGCGTAATTGTTGATC
25	H982A+H983A+H985A-F	AATTACGCCGCTGCGGCTGATGCCTACCTGAAT
26	H982A+H983A+H985A-R	ATTCAGGTAGGCATCAGCCGCAGCGGCGTAATT
27	H982A+H983D+H985A-F	GATCAACAATTACGCCGATGCGGCTGATGCCTAC
28	H982A+H983D+H985A-R	GTAGGCATCAGCCGCATCGGCGTAATTGTTGATC
29	D986A-F	TACCACCATGCGCATGCGGCCTACCTGAATGCA
30	D986A-R	TGCATTCAGGTAGGCCGCATGCGCATGGTGGTA

Table S2. The SpCas9 mutants

Single-point mutants	Double-point mutants	Triple-point mutants
E762A	H982A+H983A	H982A+H983A+H985A
D839A	H982A+H985A	H982A+H983D+H985A
N863A	H983A+H985A	
H982A	H982A+H983D	
H983A		
H983D		
H985A		
D986A		
H840A		

Table S3. The base sequences of SpCas9

Base sequences (5'→3') of SpCas9 template strand
GGCGACAAGAAGTACTCCATTGGGCTCGATATCGGCACAAACAGCGTCGGCTGGGCCGTCA
TTACGGACGAGTACAAGGTGCCGAGCAAAAAATTCAAAGTTCTGGGCAATACCGATCGCCA
CAGCATAAAGAAGAACCTCATTGGCGCCCTCCTGTTCGACTCCGGGGAGACGGCCGAAGCC
ACGCGGCTCAAAAGAACAGCACGGCGCAGATATACCCGCAGAAAGAATCGGATCTGCTACC
TGCAGGAGATCTTTAGTAATGAGATGGCTAAGGTGGATGACTCTTTCTTCCATAGGCTGGA
GGAGTCCTTTTTGGTGGAGGAGGATAAAAAGCACGAGCGCCACCCAATCTTTGGCAATATC
GTGGACGAGGTGGCGTACCATGAAAAGTACCCAACCATATATCATCTGAGGAAGAAGCTTG
TAGACAGTACTGATAAGGCTGACTTGCGGTTGATCTATCTCGCGCTGGCGCATATGATCAA
ATTTCGGGGACACTTCCTCATCGAGGGGGACCTGAACCCAGACAACAGCGATGTCGACAAA
CTCTTTATCCAACTGGTTCAGACTTACAATCAGCTTTTCGAAGAGAACCCGATCAACGCAT
CCGGAGTTGACGCCAAAGCAATCCTGAGCGCTAGGCTGTCCAAATCCCGGCGGCTCGAAAA
CCTCATCGCACAGCTCCCTGGGGAGAAGAAGAACGGCCTGTTTGGTAATCTTATCGCCCTG
TCACTCGGGCTGACCCCCAACTTTAAATCTAACTTCGACCTGGCCGAAGATGCCAAGCTTC
AACTGAGCAAAGACACCTACGATGATGATCTCGACAATCTGCTGGCCCAGATCGGCGACCA
GTACGCAGACCTTTTTTTGGCGGCAAAGAACCTGTCAGACGCCATTCTGCTGAGTGATATT
CTGCGAGTGAACACGGAGATCACCAAAGCTCCGCTGAGCGCTAGTATGATCAAGCGCTATG
ATGAGCACCACCAAGACTTGACTTTGCTGAAGGCCCTTGTCAGACAGCAACTGCCTGAGAA
GTACAAGGAAATTTTCTTCGATCAGTCTAAAAATGGCTACGCCGGATACATTGACGGCGGA
GCAAGCCAGGAGGAATTTTACAAATTTATTAAGCCCATCTTGGAAAAAATGGACGGCACCG
AGGAGCTGCTGGTAAAGCTTAACAGAGAAGATCTGTTGCGCAAACAGCGCACTTTCGACAA
TGGAAGCATCCCCCACCAGATTCACCTGGGCGAACTGCACGCTATCCTCAGGCGGCAAGAG
GATTTCTACCCCTTTTTGAAAGATAACAGGGAAAAGATTGAGAAAATCCTCACATTTCGGA
TACCCTACTATGTAGGCCCCCTCGCCCGGGGAAATTCCAGATTCGCGTGGATGACTCGCAA
ATCAGAAGAGACCATCACTCCCTGGAACTTCGAGGAAGTCGTGGATAAGGGGGCCTCTGCC
CAGTCCTTCATCGAAAGGATGACTAACTTTGATAAAAATCTGCCTAACGAAAAGGTGCTTC
CTAAACACTCTCTGCTGTACGAGTACTTCACAGTTTATAACGAGCTCACCAAGGTCAAATA
CGTCACAGAAGGGATGAGAAAGCCAGCATTCCTGTCTGGAGAGCAGAAGAAAGCTATCGTG
GACCTCCTCTTCAAGACGAACCGGAAAGTTACCGTGAAACAGCTCAAAGAAGACTATTTCA
AAAAGATTGAATGTTTCGACTCTGTTGAAATCAGCGGAGTGGAGGATCGCTTCAACGCATC
CCTGGGAACGTATCACGATCTCCTGAAAATCATTAAAGACAAGGACTTCCTGGACAATGAG
GAGAACGAGGACATTCTTGAGGACATTGTCCTCACCCTTACGTTGTTTGAAGATAGGGAGA
TGATTGAAGAACGCTTGAAAACTTACGCTCATCTCTTCGACGACAAAGTCATGAAACAGCT
CAAGAGGCGCCGATATACAGGATGGGGGCGGCTGTCAAGAAAACTGATCAATGGGATCCGA
GACAAGCAGAGTGGAAAGACAATCCTGGATTTTCTTAAGTCCGATGGATTTGCCAACCGGA
ACTTCATGCAGTTGATCCATGATGACTCTCTCACCTTTAAGGAGGACATCCAGAAAGCACA
AGTTTCTGGCCAGGGGGACAGTCTTCACGAGCACATCGCTAATCTTGCAGGTAGCCCAGCT
ATCAAAAAGGGAATACTGCAGACCGTTAAGGTCGTGGATGAACTCGTCAAAGTAATGGGAA
GGCATAAGCCCGAGAATATCGTTATCGAGATGGCCCGAGAGAACCAAACTACCCAGAAGGG
ACAGAAGAACAGTAGGGAAAGGATGAAGAGGATTGAAGAGGGTATAAAAGAACTGGGGTCC
CAAATCCTTAAGGAACACCCAGTTGAAAACACCCAGCTTCAGAATGAGAAGCTCTACCTGT
ACTACCTGCAGAACGGCAGGGACATGTACGTGGATCAGGAACTGGACATCAATCGGCTCTC

Base sequences (5'→3') of SpCas9 template strand (continued Table S3)
CGACTACGACGTGGATCATATCGTGCCCCAGTCTTTTCTCAAAGATGATTCTATTGATAAT
AAAGTGTTGACAAGATCCGATAAAAATAGAGGGAAGAGTGATAACGTCCCCTCAGAAGAAG
TTGTCAAGAAAATGAAAAATTATTGGCGGCAGCTGCTGAACGCCAAACTGATCACACAACG
GAAGTTCGATAATCTGACTAAGGCTGAACGAGGTGGCCTGTCTGAGTTGGATAAAGCAGGC
TTCATCAAAAGGCAGCTTGTTGAGACACGCCAGATCACCAAGCACGTGGCCCAAATTCTCG
ATTCACGCATGAACACCAAGTACGATGAAAATGACAAACTGATTCGAGAGGTGAAAGTTAT
TACTCTGAAGTCTAAGCTGGTCTCAGATTTCAGAAAGGACTTTCAGTTTTATAAGGTGAGA
GAGATCAACAATTACCACCATGCGCATGATGCCTACCTGAATGCAGTGGTAGGCACTGCAC
TTATCAAAAAATATCCCAAGCTTGAATCTGAATTTGTTTACGGAGACTATAAAGTGTACGA
TGTTAGGAAAATGATCGCAAAGTCTGAGCAGGAAATAGGCAAGGCCACCGCTAAGTACTTC
TTTTACAGCAATATTATGAATTTTTTCAAGACCGAGATTACACTGGCCAATGGAGAGATTC
GGAAGCGACCACTTATCGAAACAAACGGAGAAACAGGAGAAATCGTGTGGGACAAGGGTAG
GGATTTCGCGACAGTCCGGAAGGTCCTGTCCATGCCGCAGGTGAACATCGTTAAAAAGACC
GAAGTACAGACCGGAGGCTTCTCCAAGGAAAGTATCCTCCCGAAAAGGAACAGCGACAAGC
TGATCGCACGCAAAAAAGATTGGGACCCCAAGAAATACGGCGGATTCGATTCTCCTACAGT
CGCTTACAGTGTACTGGTTGTGGCCAAAGTGGAGAAAGGGAAGTCTAAAAAACTCAAAAGC
GTCAAGGAACTGCTGGGCATCACAATCATGGAGCGATCAAGCTTCGAAAAAAACCCCATCG
ACTTTCTCGAGGCGAAAGGATATAAAGAGGTCAAAAAAGACCTCATCATTAAGCTTCCCAA
GTACTCTCTCTTTGAGCTTGAAAACGGCCGGAAACGAATGCTCGCTAGTGCGGGCGAGCTG
CAGAAAGGTAACGAGCTGGCACTGCCCTCTAAATACGTTAATTTCTTGTATCTGGCCAGCC
ACTATGAAAAGCTCAAAGGGTCTCCCGAAGATAATGAGCAGAAGCAGCTGTTCGTGGAACA
ACACAAACACTACCTTGATGAGATCATCGAGCAAATAAGCGAATTCTCCAAAAGAGTGATC
CTCGCCGACGCTAACCTCGATAAGGTGCTTTCTGCTTACAATAAGCACAGGGATAAGCCCA
TCAGGGAGCAGGCAGAAAACATTATCCACTTGTTTACTCTGACCAACTTGGGCGCGCCTGC
AGCCTTCAAGTACTTCGACACCACCATAGACAGAAAGCGGTACACCTCTACAAAGGAGGTC
CTGGACGCCACACTGATTCATC
